# Expression of HA of HPAI H5N1 Virus at US2 Gene Insertion Site of Turkey Herpesvirus Induced Better Protection than That at US10 Gene Insertion Site

**DOI:** 10.1371/journal.pone.0022549

**Published:** 2011-07-27

**Authors:** Hongbo Gao, Hongyu Cui, Xianlan Cui, Xingming Shi, Yan Zhao, Xiaoyan Zhao, Yanming Quan, Shuai Yan, Weiwei Zeng, Yunfeng Wang

**Affiliations:** 1 Division of Avian Infectious Diseases, State Key Laboratory of Veterinary Biotechnology, Harbin Veterinary Research Institute, The Chinese Academy of Agricultural Sciences, Harbin, China; 2 Department of Animal Medicine, College of Animal Science and Veterinary Medicine, Hebei North University, Zhang-Jia-Kou, China; 3 Animal Health Laboratory, Department of Primary Industries, Parks, Water and Environment, Prospect, Australia; Erasmus Medical Center, The Netherlands

## Abstract

Herpesvirus of turkey (HVT) is being widely used as a vector for development of recombinant vaccines and US2 and US10 genes are often chosen as insertion sites for targeted gene expression. However, the different effects of the two genes for generation of recombinant HVT vaccines were unknown. In order to compare the effects of inserted genes in the two sites on the efficacy of the recombinant vaccines, host-protective haemagglutinin (HA) gene of the highly pathogenic avian influenza virus (HPAIV) H5N1 was inserted into either US2 or US10 gene locus of the HVT. The resulting US2 (rHVT-US2-HA) or US10 (rHVT-US10-HA) recombinant HVT viruses were used to infect chicken embryo fibroblasts. Plaques and the growth kinetics of rHVT-US2-HA-infected chicken embryo fibroblasts were similar to those of parental HVT whereas rHVT-US10-HA infected chicken embryo fibroblasts had different growth kinetics and plaque formation. The viremia levels in rHVT-US10-HA virus-infected chickens were significantly lower than those of rHVT-US2-HA group on 28 days post infection. The vaccine efficacy of the two recombinant viruses against H5N1 HPAIV and virulent Marek's disease virus was also evaluated in 1-day-old vaccinated chickens. rHVT-US2-HA-vaccinated chickens were better protected with reduced mortality than rHVT-US10-HA-vaccinated animals following HPAIV challenge. Furthermore, the overall hemaglutination inhibition antibody titers of rHVT-US2-HA-vaccinated chickens were higher than those of rHVT-US10-HA-vaccinated chickens. Protection levels against Marek's disease virus challenge following vaccination with either rHVT-US2-HA or rHVT-US10-HA, however, were similar to those of the parental HVT virus. These results, for the first time, indicate that US2 gene provides a favorable foreign gene insertion site for generation of recombinant HVT vaccines.

## Introduction

Herpesvirus of turkey (HVT) is a naturally occurring, non-pathogenic alphaherpesvirus originally isolated from domestic turkeys in the late of 1960s [1]. HVT is a member of the genus *Mardivirus* and is antigenically and genetically related to Marek's disease (MD) virus (MDV), the etiologic agent of the globally and economically significant Marek's disease in chickens [2], [3]. MDV is a chicken pathogen that results in the development of T-cell lymphomas and mononuclear infiltration of peripheral nerves in a matter of weeks following infection [2]. Since antigenic similarities between MDV and HVT have been documented, these similarities have been exploited in the context of vaccination strategies, that is, HVT vaccination of chickens has resulted in long-lasting, protective immunity against MD [4], [5]. Since the early 1970s chicken vaccinations with HVT have dramatically reduced MD-related losses [6].

HVT not only serves as a viable vaccine option for prevention of MD but can also be used as a vector for development of recombinant vaccines. Specifically, HVT provides an efficient delivery system for immunogenic genes that can facilitate the control of multiple poultry-related diseases. HVT possesses some ideal characteristics: (1) HVT is a herpesvirus that infects chickens persistently, resulting in continuous immune system stimulation that helps maintain protective antibody levels elevated, (2) HVT vaccine is also available in a cell-free ‘dry’ (lyophilized) form that is convenient for long-term storage and transport [7], [8] and (3) MDV genome is large enough to accommodate multiple foreign genes. Recombinant HVT (rHVT) vaccine has been proven to be one of useful viral vectors of targeted gene expression and developed for the prevention of diseases caused by infections with various fowl disease-associated viruses [7], [9]–[13].

Some genes in some alphaherpesviruses have been reported as ‘nonessential’ for viral growth in cell culture, but ‘nonessential’ genes can be used in the context of specific *in vitro* systems and do not necessarily suggest that a respective gene product is nonessential in all *in vitro* or *in vivo* models. Nevertheless, nonessential genes are usually the targets of foreign gene insertions for design of alphaherpesvirus vectors [1], [10]. In the context of the herpesvirus genome, the unique short (US) 1, US2, US10 and thymidine kinase genes have been defined as ‘nonessential’ for growth in cell cultures [11], [14], [15] and the US2 and US10 genes have been used as insertion sites for foreign genes in development of recombinant HVT or MDV. For example, when a recombinant CVI-988 (rCVI-988) expressing infectious bursal disease virus (IBDV) VP2 at the US2 site was engineered, vaccination with this recombinant vaccine conferred partial protection against virulent IBDV (>55%) and full protection against vvMDV challenge [16]. Baigent *et al.* constructed a full-length infectious bacterial artificial chromosome (BAC) clone consisting of HVT (HVT-BAC) following insertion into the US2 locus and these HVT-BAC clones conferred 100% protection against vMDV challenge [1]. In addition, rHVT expressing Newcastle disease virus (NDV) fusion protein (F) at the US10 site conferred 90% protection against velogenic NDV and effective protection against vvMDV in various studies [10], [17]. In these studies US2 and US10 gene loci were often chosen as insertion sites and the results demonstrated that insertion of foreign genes into the US2 and US10 gene loci did not impair recombinant virus replication rates *in vivo* [10], [18], [19]. Although US2 and US10 genes have been used in turn as insertion sites for generating vaccines against the same or various diseases, the different effects of the two genes for the generation of rHVT vaccines were unknown. Therefore, our goal was to find which gene, US2 or US10, would be a more suitable insertion site for foreign genes in the development of recombinant alphaherpesvirus vaccines.

Avian influenza (AI) is a highly contagious, re-emerging infectious disease affecting poultry worldwide. Highly pathogenic avian influenza (HPAI) viruses (HPAIV) are comprised of a particular avian influenza virus (AIV) subtype H5 and H7 by the World Organization for Animal Health (OIE) [20]. Conventional inactivated vaccines have been considered to be effective in the control and prevention of avian influenza outbreaks but the difficulty in differentiating infected birds from vaccinated ones limits their use [21]. The basis of protective humoral responses is contingent on the development of neutralizing antibodies against the haemagglutinin protein and a variety of vaccines under development derived from the AIV HA gene product, including recombinant virus vaccines and DNA vaccines, have shown effective protection against challenge with homologous strains [22]–[25].

In this study, we describe construction of two rHVT viruses expressing the AIV H5 HA gene at the US2 and US10 sites, respectively. The abilities of the rHVTs to replicate *in vitro* and *vivo* and elicit protective immunity in chickens following challenge with either AIV or MDV were assessed. We demonstrated that the HVT-based recombinant vaccine expressing the AIV HA gene at the US2 site conferred more effective protection against challenge with AIV when compared to protection conferred by rHVT expressing the AIV HA gene at the US10 site.

## Results

### Construction and purification of recombinant viruses

rHVT viruses expressing the AIV H5 gene at either US2 or US10 site were generated by constructing two recombinant plasmids following a series of functional fragment insertions containing the left-and right-side homologies of US2 or US10 gene in addition to both the Eco-*gpt* cassette (for selection purposes) and the HA cassette. Transfection of chicken embryo fibroblasts (CEFs) with wild-type HVT (wtHVT) DNA together with pGAB-*gpt*-HA or pUAB-*gpt*-HA, respectively, resulted in viral replication demonstrating the viability of the respective recombinant viruses. After eight rounds of selection process in selection medium, purified rHVT-US2-HA and rHVT-US10-HA recombinants were obtained, respectively.

### Southern blotting hybridization

**Figure 1:**
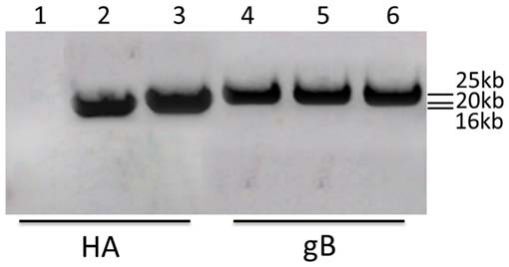
Southern blotting hybridization of BamHI-digested DNA extracted from cells infected with rHVT-US2-HA, rHVT-US10-HA or wtHVT virus. HA specific band was detected with HA probe in the DNA from rHVT-US2-HA (Lane 2) or rHVT-US10-HA (Lane 3). In contrast, no band was dectected in the DNA from wtHVT (Lane 1). With gB probe, DNA of cells infected with rHVT-US2-HA, rHVT-US10-HA or wtHVT virus all contained gB specific band (Lane 4–6).

A major 16 kb fragment from the *Bam*HI-digested rHVT-US2-HA DNA and another major 20 kb fragment from the *Bam*HI-digested rHVT-US10-HA DNA were detected with HA probe by Southern blotting hybridization, respectively, indicating that the transfer vectors were correctly inserted in the US2 region or the US10 region. We also carried out Southern hybridization to confirm the existence of gB from the wtHVT and recombinant viruses. As expected, a single band of the predicted size of about 25 kb was detected in DNA extracted from rHVT-US2-HA or rHVT-US10-HA-infected cells. A similar-size band was also detected in the DNA samples extracted from wtHVT-infected cells (Figure 1).

### Immunofluorescence staining and Western blot analysis

**Figure 2:**
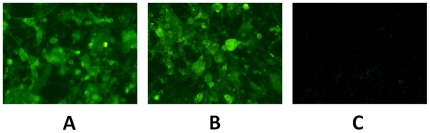
Immunofluorescence staining of cells expressing HA antigen of the highly pathogenic avian influenza virus H5N1 in turkey herpesvirus. Confluent CEFs were infected with either (A) rHVT-US2-HA, (B) rHVT-US10-HA or (C) wtHVT. Cells were incubated with polyclonal chicken antiserum against HA antigen H5 of avian influenza virus, stained with rabbit anti-chicken IgG-FITC conjugate and then examined under fluorescence microscopy 48 hours after infection. Magnification 20×.

CEFs were infected with either rHVT-US2-HA, rHVT-US10-HA or wtHVT virus. HA expression was detected by immunofluorescence staining using chicken anti-H5 serum and fluorescein isothiocyanate (FITC)-labeled rabbit anti-chicken IgG. Fluorescence was detected in recombinant virus-infected cells following microscopic analysis in contrast to cells infected with wtHVT (Figure 2). These results indicated that rHVT-US2-HA and rHVT-US10-HA were successfully expressed in CEFs.

**Figure 3:**
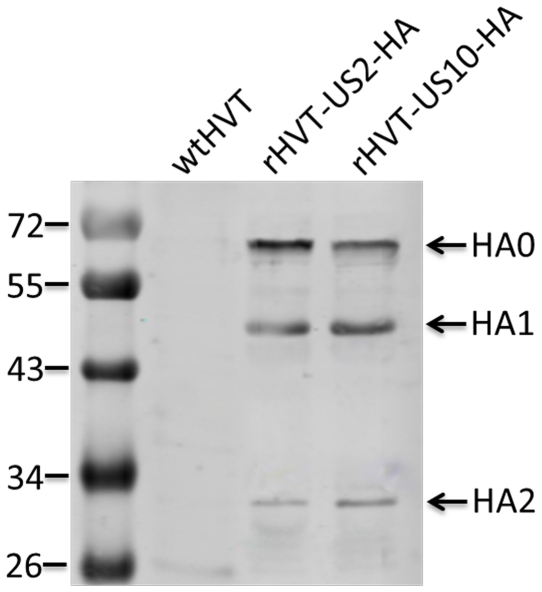
Western blot analysis. CEFs inoculated with recombinant HVT vaccines were subjected to SDS-PAGE followed by transfer onto a nitrocellulose membrane. Blots were incubated with chicken anti-HA antiserum and rabbit anti-chicken IgG conjugate. HA-specific bands corresponding to the cleaved HA1 and HA2 were detected in preparations of rHVT-US2-HA or rHVT-US10-HA-infected cells, but not wtHVT. No staining was observed after incubating blots with conjugate only (data not shown).

Three bands were detected by Western blot analysis using chicken anti-H5 AIV HA serum and IR dye 800-labeled rabbit anti-chicken IgY in lysates of cells infected with rHVT-US2-HA or rHVT-US10-HA after incomplete treatment with trypsin, which represented the intact HA precursor HA0, the cleaved products HA1 and HA2. As expected, HA specific band was not detected in CEFs infected with wtHVT (Figure 3).

### Haemagglutination assay

Haemagglutination assays using 0.5% chicken red blood cells demonstrated that the HA antigen produced on each of the two recombinant viruses agglutinated chicken red blood cells in contrast to wtHVT. The haemagglutination titers of rHVT-US2-HA, rHVT-US10-HA and wtHVT were 3log_2_, 2log_2_ and 0, respectively.

### Biological characterization of the recombinant viruses

After recombinant viruses were confirmed to express HA, we determined whether the growth curves of the US2 and US10 gene-deleted recombinant viruses were comparable to that of wtHVT. This was carried out three times by infecting CEFs with 100 plaque forming units (p.f.u.) of either wtHVT or one of the two recombinant viruses and assessing plaque formation at 0, 24, 36, 48, 54, 72, 96 and 120 h post infection.

**Figure 4:**
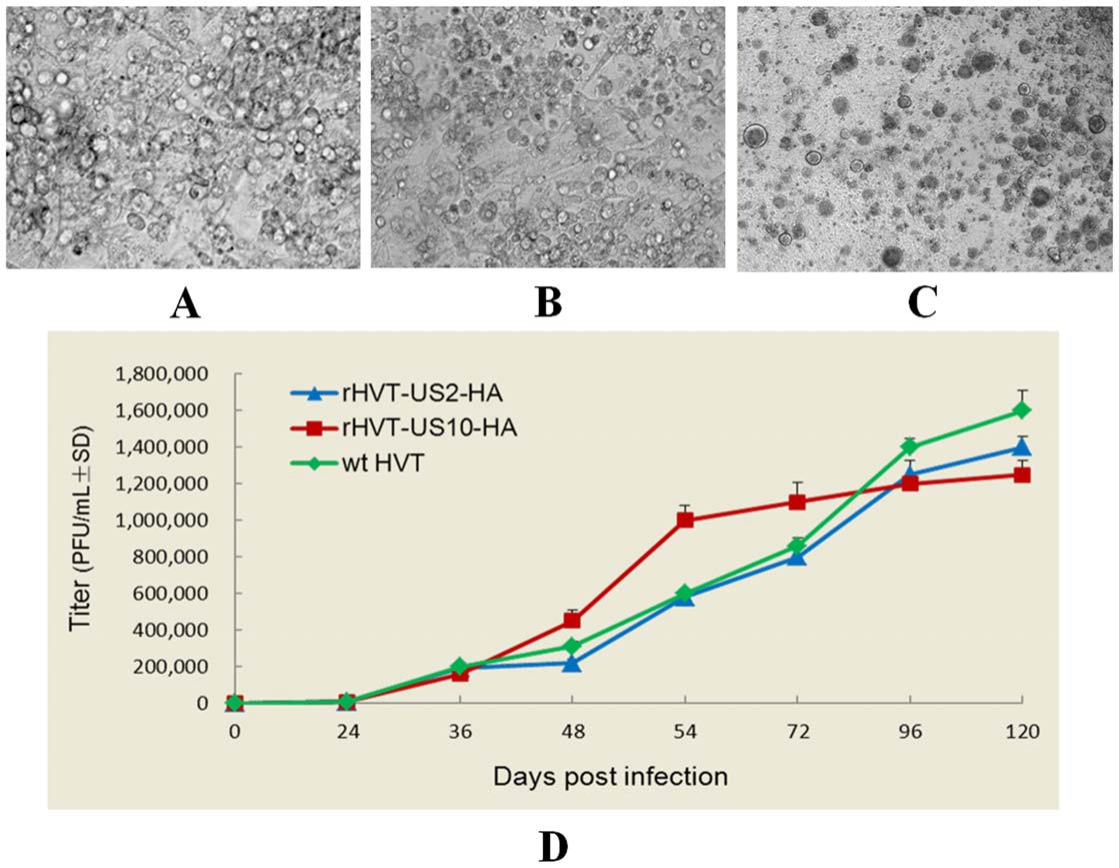
Plaque morphology and growth rates. CEFs were incubated with either (A) wtHVT, (B) rHVT-US2-HA or (C) rHVT-US10-HA for 96 h and their respective morphology was assessed. The growth rates of the recombinant and wild-type viral isolates were compared over time (D).

Plaque morphology and sizes of wtHVT and rHVT-US2-HA virus infected CEFs were indistinguishable from each other at 96 h post infection, and rHVT-US2-HA viruses had very similar *in vitro* replication kinetics to wtHVT. However, extensive syncytia were formed in HVT-US10-HA infected cells and it replicated at a higher rate than wtHVT between 48–54 h post infection and grew slowly 54 h post infection (Figure 4).

### Viremia levels of chickens infected with HVT-US2-HA or HVT-US10-HA

**Figure 5:**
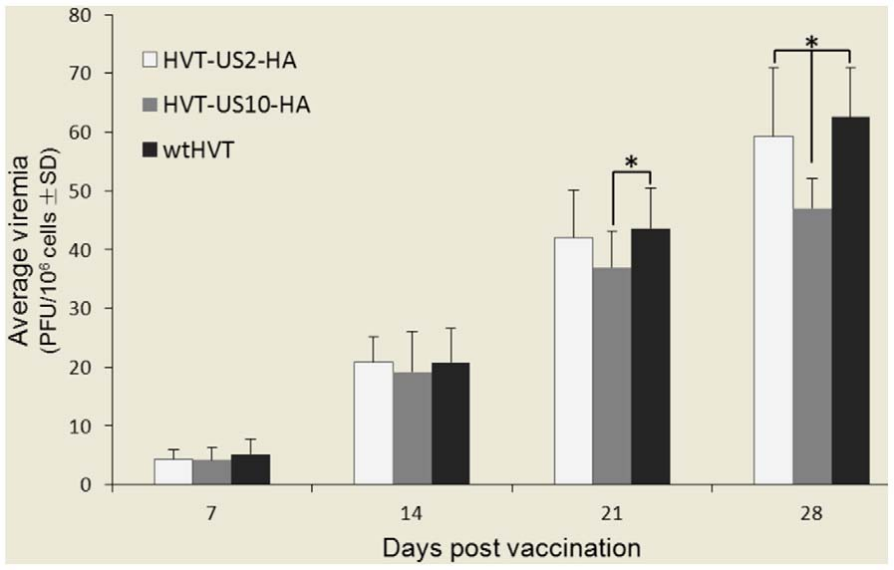
Comparison of viremia levels between rHVT-US2-HA, rHVT-US10-HA or wtHVT. Day old chicks were vaccinated with either wtHVT, rHVT-US2-HA or rHVT-US10-HA and bled on 7, 14, 21, 28 days post infection for determination of viremia. Stars indicate that the differences were significant between groups (P<0.05).

The viremia levels in five birds from each group were determined on 7, 14, 21, 28 days post infection. As indicated in Figure 5, the viremia levels in rHVT-US10-HA virus-infected chickens were slightly lower than those of rHVT-US2-HA group on 14 and 21 days post infection, but were significantly lower than those of rHVT-US2-HA group on 28 days post infection (P<0.05). The viremia levels were indistinguishable between wtHVT virus infected chickens and rHVT-US2-HA virus infected chickens during the whole experimental period.

### Evaluation of protection against AIV challenge

To determine the protective efficacy of the recombinant viruses against challenge with HPAIV, chickens were infected with 10^5^ EID_50_ of HPAIV H5N1 A/Goose/HLJ/QFY/2003 4 weeks post vaccination. Only 4/15 (26.7%) chickens vaccinated with rHVT-US10-HA were protected whereas 9/15 (60%) of the chickens vaccinated with rHVT-US2-HA were protected (Table 1). In contrast to the vaccine groups, control chickens injected with wtHVT died within two days post challenge. Furthermore, we were able to isolate viruses from chickens vaccinated with rHVT-US2-HA or rHVT-US10-HA on 3 and 5 days post challenge but virus shedding was undetectable at 1 week post challenge.

**Table 1:** Protective efficacy of the recombinant vaccines against HPAIV H5 challenge in chickens.

Vaccine formulation tested	Virus isolated from collected swabs (shedding/total [log10 EID_50_])^a^				
	Day 3 p.c.		Day 5 p.c.		Survival/total
	Oropharyngeal	Cloacal	Oropharyngeal	Cloacal	
rHVT-US2-HA	4/13(2.4±0.7)	1/13(2.8±0.4)	1/10(2.1±0.2)	1/10(1.8±0.3)	9/15^A^
rHVT-US10-HA	4/9(2.1±0.3)	5/9(1.5±0.5)	3/7(2.8±0.3)	2/7(2.4±0.6)	4/15^B^
HVT Fc 126	—b	—b	—b	—b	0/15

a Oropharyngeal and cloacal swabs were collected on days 3, 5, and 7 post challenge and titrated in SPF eggs. All control group chickens died before day 7. No virus was detected in the vaccinated chickens. For this reason day 7 data are excluded.

b All chickens in this group died before day 3.

Different uppercase superscript letters indicate a significant difference (P<0.05) between groups on respective rows.

HA antibodies constitute one of the major defenses against viral infections, therefore, we examined the relative capacities of the rHVT-HA vaccines to elicit protective humoral immune responses (Table 2). Although chickens vaccinated with either rHVT-US2-HA or rHVT-US10-HA elicited HA antibody responses 2 weeks post vaccination, the antibody levels detected were low. However, haemagglutination inhibition (HI) antibody titers increased 3–4 weeks post vaccination and the increase of mean HI antibody titers in chickens vaccinated with rHVT-US2-HA was statistically significant (P<0.05) when compared to chickens vaccinated with rHVT-US10-HA on 21 and 28 days post challenge. The control group inoculated with wtHVT showed no evidence of HI antibody responses.

**Table 2:** Results of haemagglutination inhibition test of chickens vaccinated with recombinant vaccines.

Vaccine formulation tested	Log_2_ HI titer at different days post-vaccination(mean±SD)				
	7	14	21	28	35
rHVT-US2-HA	0	1.6^A^±0.5	3.05^A^±0.61	4.05^A^±0.69	14.3±1.11
rHVT-US10-HA	0.3±0	0.9^B^±0.3	2.2^B^±0.35	3.15^B^±0.70	13.5±1.04
HVT Fc 126	ND	ND	ND	ND	—a

ND = not determined.

a Negative control; all died.

Different uppercase superscript letters indicate a significant difference (P<0.05) between groups on respective rows.

### Evaluation of protection against MDV challenge

**Figure 6:**
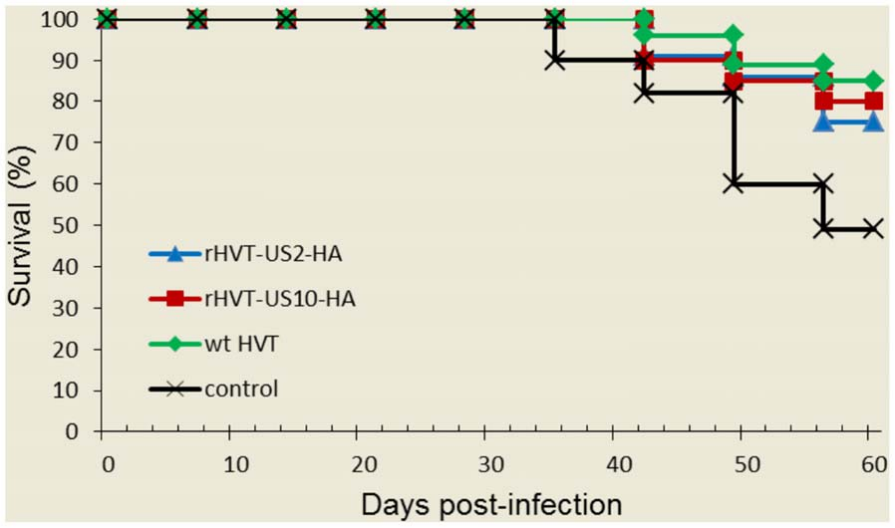
Characterization of vaccine efficacy. Day old chickens were vaccinated with either wtHVT, rHVT-US2-HA or rHVT-US10-HA and challenged 14 days later with MDV J-1. Data are expressed as percentage survival of birds from unvaccinated or vaccinated groups.

The protective efficacy against Marek's disease of the rHVT vaccines was determined by assessing cumulative survival rates and gross/histological lesions in vaccinated animals post challenge with MDV. Evidence of MD was observed in control animals 4 weeks post challenge with vMDV strain J-1and 95% of the chickens in this group developed MD during the 60-day experimental period (Table 3). Lymphoid tumors were observed in several visceral organs of the dead chickens, particularly in hearts and kidneys. In contrast, the vaccine groups had survival rates above 70% (Figure 6); 20% of wtHVT and rHVT-US10-HA vaccinated animals presented with MD (PI value of 78.9) and rHVT-US2-HA vaccinated animals had an MD incidence rate of 33% (PI value of 65.3). The PI values of the three vaccine groups were not statistically different (P>0.05).

**Table 3:** Protective efficacy of recombinant HVTs and wtHVT against MDV challenge.

Vaccine	Number of chickens/group	MD mortality	MD%	Protection Index (PI) %
rHVT-US2-HA	15	4	33	65.3
rHVT-US10-HA	15	3	20	78.9
HVT Fc126	15	3	20	78.9
Control	20	11	95	0

MD (%) indicates the percentage of MDV-infected chickens that died after challenge with MDV strain vJ-1 or developed gross tumors prior to experimental termination.

## Discussion

In this study, two recombinant HVTs (rHVT-US2-HA and rHVT-US10-HA) were generated, and preliminary experiments indicated that the respective insertions did not affect the rHVT growth in CEFs as previously described [10], [19]. Results from *in vitro* viral growth curve analysis demonstrated that replication rates and plaque sizes of rHVT-US2-HA infected CEFs were similar to those of parental HVT. In contrast, plaque morphology and replication rates of rHVT-US10-HA infected CEFs were different to those of wtHVT [10]. Our *in vivo* experiments have shown the different growth rates of the recombinant HVT viruses and wtHVT in chickens by viremia analysis. The viremia levels in rHVT-US10-HA virus-infected chickens were significantly lower than those of rHVT-US2-HA group on 28 days post infection and there was no obvious difference on growth rate between rHVT-US2-HA and wtHVT group, revealing a similar *in vitro* growth rate. However, rHVT-US10-HA replicated at a higher rate than wtHVT between 48–54 h post vaccination, indicating a different *in vivo* growth rate. The viremia levels in rHVT-US10-HA group were lower than those in rHVT-US2-HA group or wtHVT group during the whole experimental period.

The virus challenge experiments showed that rHVT-US2-HA and rHVT-US10-HA vaccinated chickens were 60 and 26.7% protected against HPAIV challenge and virus shedding data were consistent with mortality, respectively. Serological data suggested that rHVT-vaccinated chickens persistently stimulated their host-immune systems. Chickens vaccinated with rHVT-US2-HA had significantly higher HI antibody titer levels compared to rHVT-US10-HA vaccinated chickens on 21 and 28 days post infection. These results indicated that HVT expressing the HA gene at the US2 position was significantly more effective in conferring protective immunity than the virus expressing HA at the US10 site.

Both rHVTs conferred effective protection against vMDV J-1 challenge. rHVT-US10-HA and parental HVT conferred the same level of protection against MDV challenge (PI value 78.9) and there were no significant differences between the two groups whereas protection conferred by rHVT-US2-HA was slightly less effective (PI value 65.3). These results demonstrated that HA insertion into either the US2 or US10 gene positions did not significantly affect the rHVT inducted protection against MDV, confirming the previous findings [1], [10].

When HVT infects CEFs, the cytopathic effect (CPE) was commonly observed first as round cells and then their fusion (syncytia) that formed cell foci. The degenerated cells eventually detached from the dish, producing plaques on the monolayer cell sheet. Previous studies showed that particular deletion mutations could alter viral plaque morphology. Mutation in gB gene of HSV-1 caused extensive syncytia formation in infected cells, rather than rarely caused cell-cell fusion as previously reported [26]. Obvious and extensive syncytia were formed after CEFs were infected with rHVT-US10-HA. It is assumed that US10 gene was responsible for plaque morphology of HVT. In previous MDV/HVT US10 gene relevant studies, the corruption of this site did not affect plaque morphology, but all these studies adopted insertion method for interruption and part of US10 gene could still be expressed [10], [27]. We replaced HVT US10 gene with targeted gene in our study, which perhaps can explain why we obtained different results. Nonetheless, we cannot rule out the possibility that HA protein expression has impact on viral plaque morphology. Additional studies are currently underway to define factors involved in mediating these effects.

Previously, recombinant fowlpox virus (rFPV) or Newcastle disease virus (rNDV) expressing the AIV HA gene were generated and vaccination data using these recombinant viruses demonstrated that the rFPV and rNDV conferred significant levels of protection against AIV challenge, indicating that recombinant viral vector expressing AIV gene was a good candidate for control of avian influenza [21], [25], [28]–[31]. In this study, the two rHVTs conferred only partial protection against AIV challenge. However, beyond our expectation, almost 90% of chickens in control group died within 24 h post challenge and the rest of them died two days post challenge. In previous studies, the cases like this high mortality of chickens in control group were rare. Therefore, the reason for the slightly lower protection levels conferred by the rHVT vaccines was likely due to either low immunization dose or high dose of challenge with HPAIV, overwhelming protective immune responses. Most of HVT-vector based antigen delivery systems have been developed for making recombinant viral vaccines. Studies on recombinant HVT vaccines have been attempted to develop as bivalent vaccines against NDV, IBDV or AIV, besides protection from MDV [7], [9], [10], [16]. Remarkably, the resulting vaccine Vaxxitek^R^ HVT+IBD was licensed and commercialized as an animal herpesvirus vector based product. Therefore, HVT-vector is a good potential candidate for vaccine development. However, current MDV vaccines including HVT cannot stop viral replication and shedding in chicken although they can protect against tumour formation and hence mortality [1]. The continued evolution of field viruses towards pathotypes of greater virulence is attributed to the selection pressures imposed on these virulent viruses in vaccinated chicken. Therefore, HVT vaccine may not effectively protect against MDV in the future.

Our study, for the first time, describes different effects of US2 and US10 insertion sites in the development of recombinant HVTs and the US2 gene locus as an insertion site for the expression of vaccine targets is more effective than US10 site in construction of an rHVT vaccine for use in chickens.

## Materials and Methods

### Ethics Statement

Animal experiments were approved by Harbin Veterinary Research Institute of the Chinese Academy of Agricultural Sciences and performed in accordance with animal ethics guidelines and approved protocols. The Animal Ethics Committee approval number was Heilongjiang-SYXK 2006-032.

### Viruses and cells

The wtHVT FC126 strain (twelfth duck embryo fibroblast passage stock) was used for construction of the recombinant viruses. MDV strain J-1 is a reference virulent MDV strain isolated from Beijing district in China [32] and is a standard virulent reference challenge strain in MDV research (tenth duck embryo fibroblast passage stock) [33], [34]. Both viral strains were obtained from the Avian Infectious Diseases Laboratory of the Harbin Veterinary Research Institute of the Chinese Academy of Agricultural Sciences and were propagated in primary CEFs prepared from 10-day-old, specific-pathogen-free embryos.

HPAIV H5N1 virus A/goose/Guangdong/3/96 [35] and A/Goose/HLJ/QFY/2003 [36] shares approximately 97% identity in HA gene. They were propagated in the allantoic cavities of 10-day-old SPF chicken embryonated eggs and then kept at −70°C before RNA extraction or use in challenge studies.

### Cloning of the AIV HA gene and HA cassette

cDNA corresponding to the A/goose/Guangdong/3/96 HA open reading frame (1,716 nucleotides) was PCR-amplified using specific primers (Table 4) that included Kozak consensus sequences [37]. The PCR products of cDNA HA were digested with *Hin*dIII and *Sal*I and cloned into the pN1-EGFP-derived pN1 vector (deficient in EGFP) (Clontech, Tokyo, Japan). The HA cassette containing the HCMV immediate-early promoter and the SV40 poly-adenylation signal were PCR-amplified using primers containing a *Pac*I restriction site (Table 4).

**Table 4:** PCR primer sequences used in amplification experiments.

Primer	Sequence	Target
HA-upper	AGGAAGCTTTACCATGGAGAGAATAGTGCTTC	HA open reading frame
HA-lower	CGGGTCGACTTAAATGCAAATTCTGCATTGTA	
HA cassette-upper	CGGCGGTTAATTAACGCCATGCATTAGTTATT	HA cassette
HA cassette-lower	CGG CGG TTAATTAACGCTTACAATTTACGCCT	
g*pt* cassette-upper1	ATTAAGCTTCGCCATGCATTAGTTATTAATAGT	g*pt* cassette with *Hin*dIII
*gpt* cassette-lower1	ATCAAGCTTCGCTTACAATTTACGCCTTAAGAT	
US10 left-upper	GAGCTCGGGTCCGGGAGGAAGTGA	2.6 kb left region
US10 left-lower	GCGGCCGCTAATCAACATATATTGTAT	
US10 right-upper	TAAGCGGCCGCTTAATTAACATAGGCACGCTCTGATGT	2.6 kb right region
US10 right-lower	CGGAAGCTTAGATTAGCAGATTTTCTGG	
*gpt* cassette-upper2	ATTGCGGCCGCCGCCATGCATTAGTTATTAATAGT	*gpt* cassette with *Not*I
*gpt* cassette-lower2	ATCGCGGCCGCCGCTTACAATTTACGCCTTAAGAT	
HA test-upper	ATGGAGAGAATAGTGCTTCTCC	HA probe
HA test-lower	CAAATTCTGCATTGTAACGAT	
gB-upper	AGGGAAAGTAGTAGTCGGGGCTGCAGGG	gB probe
gB-lower	TTCATCATCCGTCTCAGAATCCGTGTCG	

### Construction of the US2 (pGAB-gpt-HA) deletion plasmid

For construction of the US2 gene transfer vector, guanine phosphoribosyl transferase gene (Eco-*gpt*) was PCR-amplified from the pEco-*gpt* plasmid using primers containing *Hin*dIII restriction enzyme sites (Table 4) and then cloned into pGAB that included 2.0 and 2.7 kilo base pair (kb) fragments flanking the HVT US2 gene to obtain the pGAB-*gpt* plasmid [38]. The pEco-*gpt* plasmid was constructed in our laboratory, which contained the *E. coli* selective *gpt* marker under the control of the HCMV immediate-early promoter [36]. The HA cassette was cut using *Pac*I and inserted into pGAB-*gpt* to obtain the transfer plasmid pGAB-*gpt*-HA.

### Construction of the US10 deletion plasmid (pUAB-gpt-HA)

The US10 gene transfer vector was constructed by PCR-amplification of two fragments (2.6 kb and 2.6 kb) mapping to each side of the HVT US10 open reading frame using primers containing appropriate restriction enzyme sites (Table 4). Both fragments were subsequently cloned into the pUC119 vector (TaKaRa, Dalian, China) to generate pUAB. The Eco-*gpt* gene was then PCR amplified from the pEco-*gpt* plasmid using primers containing *Not*I restriction site (Table 4) and ligated with pUAB to obtain the plasmid pUAB-*gpt*. The HA cassette was then cut by *Pac*I and inserted into the pUAB-*gpt* to obtain the recombination plasmid pUAB-*gpt*-HA.

### Transfection and isolation of HVT recombinants

Recombinant HVT viruses were generated as described previously [1], [38]. Briefly, primary CEFs were co-transfected with 1 µg of each of pGAB-*gpt*-HA and 5 µg HVT DNA using liposome [39]. The transfected cells were maintained in selective medium containing mycophenolic acid (350 µg/ml), xanthine (70 µg/ml) and hypoxanthine (100 µg/ml), and monitored daily for CPE. To purify rHVT-US2-HA, virus-containing cells were passaged in selection medium for eight rounds until no cells with CPE were observed in selection medium suspensions. The rHVT-US10-HA virus was obtained by the same method.

### Southern blot analysis

Total viral (rHVT-US2-HA, rHVT-US10-HA or wtHVT) DNA was extracted using the sodium dodecyl sulfate (SDS)-protinase K-phenol protocol and analysed by restriction digestion with *Bam*HI. For Southern hybridization, DNA was separated by 1.0% agarose gel electrophoresis, transferred to membranes and probed with DIG-labeled probes (Roche Molecular Biochemicals, Mannheim, Germany) specific for glycoprotein B (gB) and HA according to the manufacturer's protocols. The sequences of the primers used to synthesize the DIG-labeled probes are shown in Table 4.

### Indirect immunofluorescence assay (IFA) and Western blot analysis

Chicken embryo fibroblast monolayers (80–90% confluent) were infected with rHVT-US2-HA, rHVT-US10-HA or wtHVT respectively, and then washed 3 times with phosphate buffered saline (PBS) and fixed with ice-cold ethanol for 15 min following the appearance of CPE. The wells were overlaid with polyclonal chicken antibodies produced by vaccination with the H5 AIV HA gene DNA vaccine (1∶100), and incubated at 37°C for 1 h. The wells were washed 3 times with PBS and incubated with anti-chicken IgY (IgG) (whole molecule)-FITC antibody produced in rabbit (1∶300) (Sigma, Shanghai, China) at 37°C for 1 h. Wells were then washed as above, dried and analyzed using an inverted phase contrast microscope with fluorescence light and a 20×ELWD objective (Nikon, Tokyo, Japan).

Western blot analysis was carried out as described previously [40]. Briefly, primary CEFs were infected by PCR-positive recombinant viruses, respectively, and cells collected when CPE was detected. Total cell lysates were prepared following incubation with lysis buffer (10 mM Tris-Cl, pH 7.4, 1 mM MgCl_2_, 0.5% NP40, 20 µg/ml DNase I), followed by 0.1% trypsin for 30 min, subjected to 10% SDS-PAGE and then transferred to nitrocellulose. For HA protein detection, membranes were incubated with chicken H5-AIV HA specific antiserum (1∶100) followed by detection with IR dye 800-labeled polyclonal rabbit anti-chicken IgG (Li-Cor, Lincoln, NE, USA) (1∶2000) and analyzed using an Odyssey infrared imager (Li-Cor). Cells infected with wtHVT were used as negtive control.

### Haemagglutination assay

CEFs were infected with rHVT-US2-HA and rHVT-US10-HA, respectively, and the rHVT expressing AIV H5 HA in the media concentrated following precipitation with 10% PEG-8000. The precipitant was resuspended in PBS for the HA assay. Chicken red blood cells were washed three times with PBS and resuspended (5%, v/v) in PBS for use in the HA assay. Haemagglutination tests were performed in 96-well round-bottom microtiter plates. To determine whether recombinant viruses possessed chicken red blood cell haemagglutination properties, 50 µl of concentrated cell supernatants and an equal volume of 0.5% chicken red blood cells were added to each well and the plates incubated for 30 min at 25°C. Chicken red blood cell suspensions were mixed and allowed to settle for 30 to 45 min. The HA titers were described as the reciprocal of the highest virus dilution with 100% HA.

### Comparison of in vitro growth rates between wtHVT and recombinant viruses

The rates of *in vitro* growth of the viruses on CEFs were studied by counting the p.f.u. at various time points. For each virus 100 p.f.u. was inoculated onto tissue-culture dishes with 2×10^6^ CEFs. At various hours post inoculation, virus-infected CEFs were harvested and serial 10-fold dilutions were added in triplicate onto the 48-well plates of CEFs. After three days, the titers of the virus at each time point were calculated from the number of p.f.u. from each of the dilutions and the growth curves of rHVT-US2-HA, rHVT-US10-HA and wtHVT determined.

### Determination of viremia

One-day-old SPF chicks were assigned to three groups of 20 chickens each and vaccinated intramuscularly with a total of 3000 p.f.u. of either rHVT-US2-HA, rHVT-US10-HA or wtHVT. After vaccination, five chickens were removed weekly from each of these groups, and the levels of HVT viremia were determined for individual chickens as reported previously [10]. Briefly, blood samples in anticoagulants were collected from five birds of each group and 5 ml blood from each bird was mixed with 5 ml RPMI 1640 medium and 3 ml Histopaque 1077 (Sigma, Shanghai, China). Samples were centrifuged at 1000× g for 30 min. The leukocytes were recovered and counted. Co-cultivations were done in duplicate by seeding 2×10^6^ leukocytes onto 60-mm plates with CEF monolayers. After 5 days, dishes were stained with crystal violet and plaques were counted.

### AIV challenge experiment

One-day-old chicks were randomly divided into groups of 15 and vaccinated intramuscularly with a total of 3000 p.f.u. of either rHVT-US2-HA or rHVT-US10-HA. Another group was inoculated with wtHVT as a negative control. Day-old chicks were vaccinated only once and then bled via wing veins weekly for five times. Chick sera were tested for antibody-mediated haemagglutination inhibition using the OIE standard method. Four-weeks post vaccination chicks in each group were challenged intranasally with 0.1 ml of 10^6^ ELD_50_ of highly pathogenic H5N1 A/Goose/HLJ/QFY/2003 virus. Oropharyngeal and cloacal swabs from respective chickens were collected on days 3, 5, and 7 post challenge for virus titration and chickens were observed for disease presentation and mortality.

### MDV challenge experiment

A total of 65 1-day-old SPF chicks were vaccinated intramuscularly with 3000 p.f.u. of either rHVT-US2-HA, rHVT-US10-HA or wtHVT. Twenty negative control chicks were inoculated with non-infected CEFs. On 14 days of age birds were challenged intra-abdominally with 1000 p.f.u. virulent MDV J-1 virus. Mortality during the course of the experiment was recorded and chickens were examined for gross MD lesions. On 60 days post challenge, all surviving birds were euthanized and examined for gross and histopathological lesions. The percentage of gross MD was calculated for each test group as the number of chickens with gross MD lesions divided by number at risk (survivors plus MD deaths)×100 [41]. Vaccinal immunity to MD was expressed as a protective index (PI) calculated as the percentage of gross MD in non-vaccinated challenged control chickens minus the percentage of gross MD in vaccinated, challenged chickens divided by the percentage of gross MD in non-vaccinated challenged control chickens and multiplied by 100.

### Statistical analyses

Comparisons of single treatment among vaccinated groups were performed using a nonparametric one-way ANOVA followed by LSD's multiple comparison post test (for Table 2). Comparison of PI and protective rate was performed using χ^2^ analysis. The analyses were done by using SPSS version 17.0 for Windows (SPSS, Chicago, IL, USA). P values less than 0.05 were considered statistically significant in all cases.
